# Genotoxic Bacteria and Oncogenic Viruses in Colorectal Cancer: Evidence, Gaps, and a Proposed Interaction Model

**DOI:** 10.3390/ijms27052272

**Published:** 2026-02-28

**Authors:** Nickolas Salazar-Ulbrich, Darling Haro-Solis, Francisco Aguayo, Claudia Quezada-Monrás, Leonardo Cárcamo, Luis Collado, Diego Carrillo-Beltrán

**Affiliations:** 1 Laboratorio de Oncovirología Molecular, Instituto de Bioquímica y Microbiología, Facultad de Ciencias, Universidad Austral de Chile, Valdivia 5110566, Chile; nickolas.salazar@alumnos.uach.cl (N.S.-U.); darling.haro@alumnos.uach.cl (D.H.-S.); 2 Laboratorio de Oncovirología, Departamento de Ciencias Biomédicas, Facultad de Medicina, Universidad de Tarapacá, Arica 1000000, Chile; fraguayog@academicos.uta.cl; 3 Laboratorio de Biología Tumoral, Instituto de Bioquímica y Microbiología, Facultad de Ciencias, Universidad Austral de Chile, Valdivia 5090000, Chile; claudiaquezada@uach.cl; 4 Millennium Institute on Immunology and Immunotherapy, Facultad de Ciencias, Universidad Austral de Chile, Valdivia 5110566, Chile; 5 Servicio de Cirugía, Hospital Base Valdivia, Valdivia 5090000, Chile; leonardo.carcamo.gruebler@uach.cl; 6 Laboratorio de Enteropatógenos Bacterianos, Instituto de Bioquímica y Microbiología, Facultad de Ciencias, Universidad Austral de Chile, Valdivia 5110566, Chile; luiscollado@uach.cl

**Keywords:** JC polyomavirus (JCPyV), *Campylobacter jejuni*, colorectal cancer, *Escherichia coli*, *Helicobacter pylori*, microbiome, Epstein–Barr virus (EBV), human papillomavirus (HPV)

## Abstract

Colorectal cancer (CRC) remains a significant global health burden, with growing evidence highlighting microbial contributions to its pathogenesis. Certain genotoxigenic bacteria, such as *Escherichia coli*, *Campylobacter jejuni*, and *Helicobacter pylori*, produce virulence factors that induce DNA damage, genomic instability, and chronic inflammation—key features of carcinogenesis. At the same time, viruses such as JC polyomavirus (JCPyV), considered potentially oncogenic, and established oncogenic viruses like Epstein–Barr virus (EBV) and human papillomavirus (HPV) have been detected in colorectal tissues and are linked to cell cycle regulation, apoptosis, and DNA repair through their viral proteins. Intriguingly, recent findings suggest that bacterial genotoxins may promote the reactivation or transcriptional activity of persistent viruses such as JCPyV and EBV, possibly through DNA damage-induced stress and activation of NF-κB- or ATM-dependent signaling pathways. Despite these advances, interactions between oncogenic viruses and bacteria within the colon microbiome remain underexplored. This review integrates current evidence and provides future perspectives for addressing potential genotoxic collaboration between bacteria and viruses that could contribute to colorectal tumorigenesis. Elucidating these interactions could reveal novel biomarkers and therapeutic targets for the prevention and treatment of CRC.

## 1. Introduction

Colorectal cancer (CRC) is one of the most pressing global health problems, ranking among the most frequently diagnosed malignancies and accounting for approximately 6% of all cancer cases worldwide [1]. According to data reported by the GLOBOCAN, in 2022, more than 1.14 million new cases were reported globally, making it the third most common cancer in terms of incidence and the second leading cause of cancer mortality [1]. Incidence rates in low-income countries are estimated at 3.3 cases per 100,000 people, while in high-income countries they exceed 9.8 cases per 100,000 people [1]. Similarly, CRC mortality reaches 2.3 deaths per 100,000 people in low-income settings and exceeds 4.4 deaths per 100,000 people in high-income countries [1]. In the report by Sung et al. (2025), CRC is increasingly affecting people aged 25 to 49, with an annual incidence increase of 2–4% in this age group, documented in 27 of the 50 countries studied [2]. This study highlights the importance of investigating the factors that influence the early onset of the disease.

Risk factors for CRC are traditionally classified as genetic and environmental. Genetic predispositions include family history, Lynch syndrome, and familial adenomatous polyposis [2,3]. Contributing environmental factors include smoking, type 2 diabetes, obesity, and alcohol consumption [3,4,5]. Both genetic and environmental factors converge on a central characteristic of cancer: genomic instability, which promotes the accumulation of mutations that drive tumour initiation and progression [3]. CRC arises through multiple signaling pathways (including chromosomal instability, mismatch repair deficiency with microsatellite instability, and CpG-island methylator phenotypes) [3], and each pathway may create distinct selective pressures that shape microbial colonization and persistence [6]. Consequently, the simple detection of a microorganism in tumor tissue does not establish causation. Rigorous inference in CRC requires (i) cellular localization to the relevant compartment (tumor epithelium versus immune infiltrate), (ii) evidence of microbial activity (transcripts, proteins, or functional readouts), (iii) temporal plausibility, and (iv) reproducibility across independent cohorts with standardized methods and control of confounders such as antibiotic exposure, inflammation, and sample handling [7,8].

Beyond these widely recognized factors, recent research has begun to highlight the importance of other emerging elements. Interactions between viruses and bacteria within the colonic microbiota represent an underexplored field that could significantly contribute to genomic instability. Although some pathogens have been investigated individually, the oncogenic potential of bacterial–viral coinfections remains largely ignored. In the following sections, we review the major genotoxic viruses and bacteria implicated in colorectal carcinogenesis, analyze their individual and combined mechanisms of action, and address a model of bacterial–viral interaction based on emerging evidence.

## 2. Involvement of Human Viruses in Colorectal Oncogenesis

In recent years, considerable efforts have been directed at elucidating the oncogenic potential of specific viruses involved in human disease. A common feature among several of these pathogens is their ability to hijack the host cell cycle to optimize viral replication, often resulting in sustained cellular proliferation and impaired apoptosis. For instance, high-risk human papillomavirus (HR-HPV) promotes cell proliferation and inhibits apoptosis through the actions of its E6 and E7 oncoproteins [9], and activates the transcription of the catalytic subunit of telomerase (hTERT), thereby contributing to cellular immortalization [10]. Similarly, Epstein–Barr virus (EBV) disrupts cell cycle regulation, inhibits apoptosis, and upregulates hTERT expression, collectively fostering a pro-oncogenic cellular environment [11,12,13]. Among the viruses under investigation, polyomaviruses have attracted growing attention. The JC polyomavirus (JCPyV), widely known for its neurotropic properties, has also been detected in gastrointestinal tissues and colorectal tumors [14,15]. Its Large T Antigen (TAg) is a multifunctional oncoprotein that binds and inactivates tumor suppressors such as p53 and Rb, thereby promoting uncontrolled cell proliferation [16]. Evidence of JCPyV DNA and protein expression in CRC samples suggests that this virus may contribute to colorectal carcinogenesis, either independently or in cooperation with other microbial factors [17]. In a complementary manner, a broad diversity of viruses that constitute the human virome may indirectly influence colorectal carcinogenesis by modulating the composition and homeostatic balance of the intestinal microbiota [18,19]. In particular, these bacteriophages may promote the depletion of bacterial populations that are beneficial to the host, thereby facilitating the establishment of intestinal dysbiosis [18,19]. This dysbiotic condition may, in turn, sustain chronic inflammatory responses and disrupt epithelial homeostasis, ultimately generating a microenvironment permissive for the initiation and progression of colon cancer [18,19]. In the following section, we explore the role of JCPyV, HR-HPV and EBV in CRC, examining epidemiological findings, molecular mechanisms, and the challenges of establishing causality in a complex microbial.

### 2.1. Polyomavirus JC and BK

JCPyV, formally designated as *Betapolyomavirus secuhominis* by the ICTV [20], is a double-stranded DNA virus that establishes a persistent, asymptomatic infection in the host [21]. Its genome is approximately 5200 base pairs in size and lacks an envelope [22]. Entry into host cells occurs via an endocytic process initiated by the virus binding to sialic acid residues on the cell’s plasma membrane [18]. According to the literature, the 5-hydroxytryptamine serotonin receptor functions as a facilitator of viral entry in glial cells [21]. Another described pathway for viral entry involves the use of extracellular vesicles released by previously infected cells, which contain viral particles that can be internalized by uninfected cells through a macropinocytosis-dependent mechanism [23]. The absence of an envelope confers resistance to the virus against adverse environmental conditions, such as temperature, pH, and desiccation [24].

The infection is thought to occur mainly in childhood, but the exact mechanisms remain uncertain [24,25,26]. Worldwide, seropositivity for JCPyV is estimated at approximately 90%, increasing with age [26]. At the colonic level, JCPyV has been detected in normal epithelial cells in 0–89% and colorectal carcinomas up to 93% [15,27,28,29] (Table 1). Furthermore, meta-analyses have demonstrated a significant correlation between the presence of the TAg protein of JCPyV and CRC, with an associated risk increase of up to eleven-fold compared with cells lacking TAg expression [17].

The genome of JCPyV comprises two coding regions, one of which encodes regulatory proteins such as TAg [22]. The other coding region corresponds to the late region, which contains genetic information for the synthesis of the structural proteins VP1, VP2, and VP3, as well as the accessory region that encodes the agnoprotein [22]. The agnoprotein plays a crucial role in regulating TAg in polyomavirus, facilitating the activation of the lytic phase of the viral cycle and contributing to virion maturation [32]. These two regions are separated by a non-coding control region (NCCR), which contains the origin of viral DNA replication and regulatory sequences for the transcription of early and late genes [22]. This region enables bidirectional gene expression via a bidirectional promoter [22]. TAg promotes cell cycle progression, increasing the number of viral copies [33]. Additionally, it induces viral genome replication through its helicase activity, which unwinds DNA at the viral genome’s origin of replication (ori) [30]. JCPyV behaves as an asymptomatic and subclinical pathogen in most infected individuals [22]. However, it can be reactivated under certain conditions, particularly in immunocompromised individuals, such as those living with HIV/AIDS or receiving immunosuppressive therapies, like natalizumab, a monoclonal antibody used in the treatment of multiple sclerosis that inhibits the migration of leukocytes across the blood–brain barrier [34].

JCPyV has been associated with various pathologies, including multiple sclerosis, Crohn’s disease, and progressive multifocal leukoencephalopathy (PML) [34]. The latter disease, PML, is characterized by a rapid decline in cognitive functions [34]. Among the symptoms, affected individuals experience difficulties with speech and walking [34]. In advanced stages, it leads to dementia, along with other neurological symptoms, as a consequence of multifocal demyelination [35]. A study has demonstrated that JCPyV can reach the colon by infecting neuroglial cells of the myenteric plexus, as it is present in the enteric glia of patients with chronic idiopathic intestinal pseudo-obstruction [35]. JCPyV has the capacity to infect various cell types, with its presence detected in the kidney, lymphocytes, colon, and brain [15,36,37,38].

There are two types of JCPyV strains, commonly referred to as archetype and prototype [39]. Compared to the archetype strain, the prototype strain exhibits a rearranged NCCR [39]. This rearrangement involves the deletion of a segment of base pairs and the duplication of a 98-base pair region, which has been associated with increased viral replication [39]. Glial cells are the primary model for studying productive infection [40]. On the other hand, lymphocytes and colonic epithelial cells exhibit a non-productive infection, characteristic of a non-permissive infection, in which viral replication is not completed [41]. This type of infection has been associated with malignant cellular transformation processes [41]. This malignant association is primarily attributed to interactions between JCPyV TAg and various tumor suppressor proteins, as well as to signaling pathways regulating cell survival and proliferation [42].

One of the most relevant interactions of JCPyV TAg is with β-catenin, a protein whose dysregulation is commonly observed in CRC [43,44,45]. It has been observed that β-catenin, through its interaction with TAg, translocates to the nucleus, thereby enhancing activation of the Wnt signaling pathway, which is known to induce cell proliferation and promote metastasis in colon cancer [43,44,45]. TAg also increases survivin levels, a protein with anti-apoptotic functions [46,47,48]. In this setting, viral proteins such as the TAg can inhibit apoptotic pathways and promote cell survival, thereby contributing to the neoplastic transformation of the host cell [46,47,48].

TAg also contributes to genomic instability by interacting with IRS-1, which promotes its nuclear translocation and sequesters RAD51, an essential component of the homologous recombination repair pathway for double-strand DNA breaks [49,50]. As a result, although ATM-mediated signaling induced by JCPyV leads to RAD51 activation, no effective enhancement of DNA repair is observed [49,51]. On the other hand, it has been demonstrated that RAD51 can enhance the transcription of JCPyV early genes through a mechanism dependent on the Nuclear Factor kappa-light-chain-enhancer of activated B cells (NF-κB) signaling pathway [52]. In a study of colon cancer cells, TAg was observed to promote cell migration and invasion by activating the AKT and MAPK signaling pathways, which are frequently altered in most cases of colon cancer [53]. Furthermore, JCPyV impairs the cellular DNA damage response through its agnoprotein, which has been shown to inhibit the repair of double-strand DNA breaks by sequestering the Ku70/Ku80 complex, an essential component of the non-homologous end joining (NHEJ) pathway [54]. This additional interference with DNA repair mechanisms reinforces the role of JCPyV in promoting genomic instability and potentially contributing to oncogenic transformation in non-permissive cells. The consistent absence of TAg expression in JCPyV-positive control tissues, together with its higher expression in immunosuppressed individuals, suggests that viral reactivation and early gene expression may represent critical steps in tumor initiation. Although methodological variability across the original studies remains a limitation, the current evidence supports further investigation into TAg as a potential biomarker and mechanistic mediator in CRC development [17].

In addition to JCPyV, another member of the Polyomaviridae family that has been investigated in the context of colorectal carcinogenesis is BK virus (BKPyV) [55]. Although structurally similar to JCPyV, sharing core viral proteins and genome organization, BKPyV differs in its primary cellular tropism and disease associations [56]. BKPyV has been detected in normal epithelial cells at a baseline prevalence of 4.1–6% and in colorectal carcinomas at frequencies ranging from 21.7% to 30% [30,31] (Table 1). BKPyV is a highly prevalent virus, with seroprevalence rates of approximately 80% in the global population, particularly among adults [55,57]. A significant difference between JCPyV and BKPyV lies in their cellular tropism. While JCPyV has been detected in various tissues, including the brain and colon, BKPyV predominantly targets renal epithelial cells and is primarily associated with nephropathy and complications in kidney transplant recipients [55]. At the same time, current evidence does not establish a definitive oncogenic role for BKPyV; its presence in tumor tissues, combined with its molecular similarity to JCPyV, warrants further investigation [56,58]. Given these observations, the oncogenic potential of BKPyV has been hypothesized to arise from the expression of its TAg, which shares structural and functional homology with the JCPyV TAg [33,59,60]. In both viruses, TAg has been shown to bind and inactivate the tumor suppressors p53 and retinoblastoma protein (pRb), thereby dysregulating the cell cycle, a key step in cellular transformation [33,59,60].

### 2.2. Human Papillomavirus

HPV plays a well-established causal role in various types of malignant neoplasms, including cervical, anal, penile, and oropharyngeal cancers. In cervical cancer, HPV is detected in over 95% of cases, with high-risk genotypes, particularly HPV16 and HPV18, being the primary drivers of malignant transformation [61,62,63,64,65,66]. In anal cancer, approximately 88% of cases are associated with HPV infection, with a notably high prevalence among immunocompromised individuals and men who have sex with men. Penile cancer shows HPV positivity in about 42% to 88% of invasive tumors, with HPV16 being the most frequently identified genotype [62,63,64,65,66].

Regarding oropharyngeal cancer, HPV has been recognized as a causative agent in approximately 60% to 70% of cases in countries such as the United States and the United Kingdom, primarily affecting the palatine tonsils and the base of the tongue [61]. Studies have reported HPV detection in 73% of palatine tonsil tumors and in 50% of tumors located at the base of the tongue, with HPV genotype 16 being the most prevalent and known for its high oncogenic potential [62]. At the colonic level, HPV has been detected in normal epithelial cells at a baseline prevalence of 14.4–35%, in adenomas with variable detection rates of 36.8–52%, and in colorectal carcinomas with frequencies ranging from 1.67% to 69% [65,66,67,68,69] (Table 2).

HPV encodes oncogenic proteins, notably E6 and E7, which play a central role in malignant transformation by deregulating the cell cycle [9]. The E6 oncoprotein induces the proteasomal degradation of the tumor suppressor p53, a key regulator of DNA damage response and apoptosis [9,72]. This degradation impairs cell cycle arrest and apoptotic signaling, thereby favoring uncontrolled cellular proliferation. Meanwhile, the E7 protein mediates the degradation of the pRb, releasing the E2F transcription factor, which in turn activates genes involved in S-phase entry and DNA replication, further promoting cellular proliferation and neoplastic transformation [73].

The presence of HPV in colorectal cancer has been reported in various studies, with a prevalence of up to 51.6% in tumor tissue samples [74,75,76]. The most frequently detected genotypes correspond to the high-risk oncogenic types, HPV16 and HPV18, identified using conventional PCR techniques or in situ hybridization with specific probes [74,75,76].

The detection of HPV in colorectal tumors raises essential questions about the mechanisms underlying its activation and oncogenicity in this anatomical site. Notably, elevated mRNA levels of the viral oncoproteins E6 and E7 have been observed under conditions of nitric oxide-induced genotoxic stress [77], suggesting that the local microenvironment may play a role in modulating viral gene expression. In this regard, the presence of genotoxic bacteria or microbial communities that can indirectly induce DNA damage could promote the transcriptional upregulation of HPV oncogenes [78].

### 2.3. Epstein–Barr Virus

The Epstein–Barr virus (EBV), also known as human herpesvirus 4, is an enveloped, double-stranded DNA virus primarily transmitted through saliva [79]. Serological studies estimate that EBV infects more than 90% of the world’s population [79]. EBV has been associated with various human neoplasms, both epithelial and lymphoid, with nasopharyngeal cancer (NPC), gastric cancer (GC), and Hodgkin lymphoma (HL) being the most established and significant associations, respectively [80,81]. EBV has been detected in normal epithelial cells at baseline, at 4.1–16.4%, and in colorectal carcinomas at 18.6–51.4% [67,82,83,84,85,86] (Table 3).

In addition, another relevant study conducted in Chinese patients with colorectal adenomas or carcinomas described that Latent membrane protein 1 (LMP1) expression was detected in about 5.4% of cases, suggesting limited but potentially relevant viral activation, given the well-established transforming capacity of LMP1 [86].

EBV classically exhibits four latency types, each characterized by the expression of a distinct set of viral proteins [87]. In CRC, latent proteins such as Epstein–Barr nuclear antigen 1 (EBNA1) and LMP1 have been detected, suggesting a latency pattern similar to type II [88]. This type of latency is characterized by modulation of protein expression associated with cell survival and proliferation [87].

A mechanistic study has shown that EBV can increase the expression of proinflammatory cytokines in primary colon epithelial cultures, linking the virus to chronic inflammation and possibly to inflammatory bowel diseases or colorectal tumorigenesis [89]. In nasopharyngeal and gastric models, distinct EBV proteins dysregulate key cellular signaling pathways. Viral proteins such as LMP1 and LMP2A play a key role in cell transformation. LMP2A activates the NF-κB signaling pathway, leading to increased survivin expression [90]. LMP1 also triggers NF-κB activation, a mechanism observed in up to 90% of cases of nasopharyngeal carcinoma [91]. Furthermore, LMP2A can promote resistance to apoptosis by activating the AKT signaling pathway, thereby increasing Bcl-2 expression [92]. LMP2A has also been shown to induce cytoplasmic accumulation and nuclear translocation of β-catenin, which activates the Wnt signaling pathway, a driver of cell proliferation and metastasis in cancer [93].

The EBNA family of dormant proteins also contributes to oncogenesis. Notably, EBNA1 enhances the transcription of *MDM2* and *MDM4,* which downregulate the tumor suppressor p53 [12]. This modulation may affect DNA damage response mechanisms and promote the survival of genetically unstable cells [12]. In summary, although multiple studies have demonstrated that EBV can modulate critical pathways involved in proliferation, apoptosis, and inflammation in nasopharyngeal and gastric models, its role in colorectal carcinogenesis remains largely unknown. This underscores the need for mechanistic studies in the colon and for definitive evidence to establish a causal relationship. A summary of the main functions of viral proteins is provided below to contextualize their potential relevance to colonic epithelial biology (Table 4).

## 3. Genotoxigenic Bacteria as Cofactors in Colorectal Carcinogenesis

Building upon the hypothesis that microbial factors can modulate viral oncogene expression, it becomes equally important to consider the direct oncogenic potential of certain bacteria. Although oncogenic viruses represent a significant risk factor, they are generally insufficient on their own to initiate complete malignant transformation. Increasing evidence points to the role of bacterial pathogens in promoting carcinogenesis, particularly within the intestinal microbiota. This microbial ecosystem comprises both commensal and pathogenic species, some of which harbor genomic islands encoding genotoxins—bacterial proteins that directly damage host DNA [94]. These genotoxins have been shown to induce genomic instability, a hallmark of early carcinogenesis that drives the accumulation of somatic mutations in otherwise normal epithelial cells [95,96]. Moreover, in established tumours, genomic instability contributes to clonal evolution and the acquisition of aggressive phenotypes through the continuous generation of genetic diversity [96]. The following sections detail the known mechanisms by which various intestinal bacteria contribute to colorectal carcinogenesis.

### 3.1. pks+ Escherichia coli

*Escherichia coli* (*E. coli*) is a Gram-negative bacterium widely recognized as one of the main foodborne enteropathogens [97]. Although *E. coli* is part of the normal intestinal microbiota and exhibits a commensal relationship with the host, specific variants or pathotypes harbour virulence factors that confer pathogenic potential and enable them to cause gastrointestinal diseases. Among these factors, the most extensively studied are strains carrying the *pks* genomic island, which encodes the biosynthetic machinery for colibactin. This hybrid polyketide–nonribosomal peptide genotoxin induces DNA double-strand breaks, genomic instability, and a characteristic mutational signature implicated in colorectal carcinogenesis [94]. These *pks+ E. coli* strains are predominantly found within the B2 phylogenetic group, known for increased virulence and enhanced ability to persist in the intestinal epithelium. Notably, up to 86% of cyclomodulin-positive *E. coli* isolated from CRC samples correspond to pks+ B2 strains, underscoring their relevance in tumor biology [98,99]. Consistent with this, pathogenic B2 and D phylogroups are more frequently detected in individuals with CRC or chronic intestinal inflammation, while commensal-associated A and B1 phylogroups predominate in healthy controls [100,101]. In terms of prevalence, *pks+ E. coli* have been detected in normal colonic mucosa at baseline frequencies of 4.4–19.3%, whereas in colorectal carcinomas their prevalence increases markedly to 9.2–55.3%, supporting a potential role in tumour initiation or progression [99,100,102,103,104] (Table 5).

While colibactin-producing *E. coli* represents the most compelling mechanistic link between bacterial genotoxicity and CRC, other genotoxins also contribute to this landscape, such as the cytolethal distending toxin (CDT) [105,106]. Colibactin is a DNA-alkylating metabolite that induces direct genetic damage by forming DNA adducts, which in turn can trigger replication-stress signaling pathways [105,106]. On the other hand, the holotoxin CDT induces double-strand DNA damage through the DNase-like activity of its active subunit, CdtB [107].

CDTs are genotoxins that are not limited to *E. coli*, as a wide variety of bacterial genera possess the operons encoding CDT, including *Campylobacter* spp. and *Helicobacter* spp. [94,95,108]. Bacterial genera have been associated with promoting tumorigenesis [94,95,108].

### 3.2. Campylobacter jejuni

Various bacterial genera harbor operons encoding CDT in their genomes, including *Campylobacter jejuni* (*C. jejuni*), a Gram-negative enterobacterium that grows microaerophilically. This characteristic complicates its cultivation, as it requires hypoxic conditions for optimal growth [109]. It has a curved rod-like shape and is relatively small [109]. It is estimated that each year, between 44 and 93 cases of *Campylobacter* infection occur per 100,000 individuals worldwide [110]. Consequently, the incidence of cases has increased over the past decade across all continents [110]. With approximately 96 million cases annually, *Campylobacter* spp. has become one of the leading causes of gastrointestinal diseases worldwide [111]. *C. jejuni* is estimated to be responsible for approximately 78% to 90% of infections caused by *Campylobacter* spp. [112,113]. Moreover, approximately 98% of *C. jejuni* isolates possess the operons required for CDT encoding [114].

Infection with *Campylobacter*, known as campylobacteriosis, primarily manifests as gastroenteritis. However, in some cases, it can lead to more severe complications such as meningitis or autoimmune diseases, including the Guillain–Barré syndrome [113]. Food contamination and the consumption of undercooked chicken meat are the primary routes of *Campylobacter* infection, with meat products representing the predominant source of transmission [115].

Following transmission, *Campylobacter* targets the intestine, where it is attracted by chemotaxis toward the mucosal layer due to the high concentration of mucins, which act as a potent chemoattractant for this bacterium [116]. The flagellum of *Campylobacter* guides the bacterium by detecting chemical signals in the environment. Additionally, it enables the bacterium to move in a helical, corkscrew-like motion, facilitating its penetration of the intestinal mucosa and subsequent invasion of colonic epithelial cells [117]. The invasion of *Campylobacter* is mediated through translocation from the apical to the basolateral side of epithelial cells, a process driven by the virulence factor HtrA. This serine protease disrupts tight junctions by cleaving key proteins, including claudin-4, claudin-8, E-cadherin, and occludin [118,119,120,121]. His translocation enables *Campylobacter* to adhere to fibronectin, thereby activating signaling cascades that are not yet fully understood. This process facilitates the invasion of epithelial cells through endocytosis [122]. Upon invading host cells, *Campylobacter* persists intracellularly within a specialized vacuole formed by the virulence factor CiaIB. This factor prevents the fusion of the endosome with the lysosome, thereby promoting bacterial survival within the host cell [123].

*C. jejuni* encodes several virulence factors, among which the CDT is particularly notable. This genotoxin can induce DNA damage in host cells. Consequently, CDT represents a potential mechanism through which *C. jejuni* may contribute to tumorigenesis, particularly in CRC [92]. In this context, CDT is a genotoxin composed of three functional subunits: CdtA, CdtB, and CdtC. CdtA and CdtC mediate toxin delivery, whereas CdtB functions as the catalytically active subunit responsible for genotoxicity due to its DNase-like activity [124]. This enzymatic activity is associated with the induction of DNA damage, which may contribute to increased genomic instability, a hallmark of the carcinogenic process [125]. Various bacterial genera encode operons for the CDT genotoxin. For instance, *Helicobacter hepaticus* CDT-positive species have been shown to promote the formation of hepatic nodules. In contrast, a catalytically inactive strain, resulting from a specific mutation in CDT, does not induce nodule formation in mouse models [108]. Furthermore, recombinant CDT from *E. coli* has been observed to cause genetic damage. In contrast, no genotoxic effect was detected when recombinant CDT harbouring a mutation in the CdtB subunit was used [126].

The CDT genotoxin can induce genetic damage, which can manifest as single- or double-strand breaks, depending on the concentration [126]. At low concentrations, CDT can induce single-strand DNA breaks, leading to cell cycle arrest at the G2/S phase [126]. Consequently, during the S phase of the cell cycle, unrepaired single-strand damage from earlier phases may be exacerbated by the collapse of stalled replication forks, leading to the generation of double-strand DNA breaks [127]. On the other hand, at high concentrations, CDT can directly induce double-strand DNA breaks, regardless of the cell cycle phase [127].

The virulence factor CDT not only induces direct genetic damage through its DNase-like activity. On the other hand, it can also cause genomic damage by activating the NF-κB signaling pathway [128,129,130]. This activation, in turn, promotes nitroxidative damage to DNA by inducing the production of reactive oxygen and nitrogen species [131].

Additionally, other virulence factors of *C. jejuni* active the NF-κB signaling pathway by upregulating proinflammatory cytokines. Among these, the virulence factor CiaD stands out, as it facilitates cellular invasion and induces cytokine production [130]. Moreover, outer membrane vesicles, which contain various *C. jejuni* virulence factors, induce the secretion of multiple proinflammatory cytokines [132,133]. Another relevant inducer of proinflammatory cytokine production in Gram-negative bacteria is LPS, a component of the outer membrane that can directly trigger cytokine production and activate caspases [134]. However, in *C. jejuni*, this component is referred to as LOS because of its shorter structure, containing fewer saccharide units than LPS. LOS is characterized by a hydrophobic lipid A anchor and a conserved internal oligosaccharide core. Additionally, its external structure is variable and lacks the O-antigen that is characteristic of LPS [135].

Consequently, the inflammatory processes induced by *C. jejuni* have also been associated with chronic inflammatory diseases of the colon. CDT has been implicated in the development of post-infectious irritable bowel syndrome [136]. This effect has been proposed to occur through a cross-reactive immune response between antibodies generated against the CdtB subunit and vinculin, a structural protein present in the cytoskeleton and adherens junctions [136]. On the other hand, the disruption of adherens junctions may facilitate the entry of commensal bacteria, such as non-pathogenic *E. coli*, through the mucosal layer, allowing direct contact with colonic epithelial cells [118]. Consequently, this process could promote dysbiosis and trigger an immune response in the colon, thereby contributing to the establishment of chronic inflammation [118,135]. It has been observed that approximately 80% of *Campylobacter* infections are caused by CDT^+^ species of *C. jejuni*, which have been associated with colon cancer due to their ability to promote colorectal tumorigenesis in murine models [95].

This species plays a key role in inducing DNA damage, contributing to genomic instability and, consequently, to alterations in host gene expression in the intestine [22]. When comparing CDT- and CDT+ species of *C. jejuni*, it was observed that CDT+ species exhibited increased cytotoxicity, inflammation, and tissue damage in the colonic mucosa. Consequently, CDT is recognized as a key virulence factor due to its role in enhancing bacterial pathogenicity [137].

Inflammation and tissue damage observed in the colonic mucosa contribute to increased genomic instability. This suggests that the pro-neoplastic characteristics of *C. jejuni* CDT+ may promote carcinogenesis [137,138]. A recent study reported that *C. jejuni* CDT+ may promote metastasis via the JAK/STAT3/MMP9 signaling pathway [139]. Surprisingly, this promotion of metastasis occurs through the activity of the WT CdtB subunit. In contrast, the mutated form of CdtB significantly reduces metastasis compared to the WT form of CDT [139]. To date, no studies have systematically assessed the prevalence of *Campylobacter jejuni* CDT+ in CRC patients, nor has CDT expression been identified in human tumor tissue. This knowledge gap represents a significant limitation for establishing a clear association between *C. jejuni*, its genotoxin, and tumor development. Consequently, studies aimed at detecting and characterizing CDT in clinical samples are critically needed to advance the understanding of the role of *C. jejuni* in colorectal carcinogenesis. Other bacteria in the colon may also promote colorectal carcinogenesis through their virulence factors. One example is *Helicobacter pylori*, which has been detected in the human colon and is classified by the IARC as a Group 1 carcinogen in GC [140,141].

### 3.3. Helicobacter pylori

*Helicobacter pylori* (*H. pylori*) is a Gram-negative, microaerophilic, rod-shaped bacterium. It possesses between four and six flagella localized at one pole of the cell, which confer efficient motility [141]. Previous studies have indicated that this bacterium, whose primary route of transmission is fecal–oral, is estimated to colonize the gastric mucosa of approximately 50% of the global population [142]. It has been widely associated with various gastric pathologies, including gastric ulcers and GC [141]. Although *H. pylori* has been primarily associated with the development of GC, its potential involvement in CRC has also been explored. *H. pylori* has been detected in normal epithelial cells baseline presence of 23.1–45.6%, adenomas, with variable detection of 30.6–48.6% and colorectal carcinomas, with up to 32.9% [143,144,145,146,147] (Table 6).

This bacterium can induce genomic damage through various virulence factors, each targeting different biological processes. Among them, the CagA virulence factor stands out for its translocation from *H. pylori* into gastric epithelial cells via a type IV secretion system, its ability to induce activation of the NF-κB signaling pathway, and to promote the release of pro-inflammatory cytokines, thereby contributing to the establishment of a chronic inflammatory process [148,149].

This sustained inflammatory response can lead to genetic damage by generating reactive oxygen and nitrogen species [148]. Furthermore, CagA interacts with the SH2 domain of the tyrosine phosphatase SHP2 as an intracellular ligand, thereby promoting activation of the MAPK signaling pathway, which plays a fundamental role in regulating cell proliferation and oncogenic processes. Consequently, the disruption of the MAPK signaling pathway induced by CagA contributes to the molecular mechanisms involved in *H. pylori*-associated oncogenesis [150]. Additionally, this virulence factor has been reported to interfere with β-catenin regulation, promoting its cytoplasmic accumulation and facilitating its translocation to the nucleus. This process activates the Wnt signaling pathway [151]. Additionally, CagA has the capacity to impair DNA damage repair through a mechanism dependent on interference with the activating phosphorylation of BRCA1 and the induction of the long non-coding RNA SNHG17, which regulates RAD51 expression [152,153]

Nevertheless, the biological activity of CagA is regulated by phosphorylation of specific sites within its EPIYA domain, mediated by host cellular proteins, primarily Src and Abl kinases [154,155]. Conversely, the protein tyrosine phosphatase SHP-1 acts as a negative regulator by dephosphorylating CagA EPIYA domains, thereby impairing the biological function of this virulence factor [156].

Another virulence factor of *H. pylori* associated with its oncogenic potential is the VacA toxin, which has been shown to increase mitochondrial membrane permeability. This alteration may trigger the release of reactive oxygen species (ROS) from mitochondria, thereby causing indirect genetic damage mediated by this bacterial protein [157].

*H. pylori* has been observed to potentially increase the risk of CRC when its virulence factors reach the intestinal tract. Several studies have demonstrated that a higher presence of antibodies directed against VacA is associated with an increased likelihood of developing CRC [158,159]. In fact, *H. pylori* has been observed to dysregulate intestinal immunity by activating the NF-κB and STAT3 signaling pathways. These pathways play a crucial role in regulating inflammation and immune responses, and their sustained activation may contribute to the development of CRC [160]. *H. pylori* has been documented to constitutively activate the STAT3 signaling pathway through the induction of interleukin-11, which may involve a chronic inflammatory process. This sustained inflammation constitutes a risk factor that increases the likelihood of developing CRC [161]. A table summarizing the main functions of bacterial proteins with genotoxic capabilities is included (Table 7).

## 4. Mechanisms of Genetic Damage and Potential Bacterial–Viral Crosstalk in Colorectal Carcinogenesis

To date, direct evidence of cooperative interactions between genotoxic bacteria and oncogenic viruses in the colorectal epithelium remains scarce. However, several conceptual and mechanistic elements support the plausibility of such crosstalk. Both bacterial genotoxins, such as colibactin and CDT, and persistent or latent viral infections such as EBV, JCPyV, or HPV have been independently associated with DNA damage, genomic instability, and deregulation of epithelial homeostasis. These shared cellular targets provide a biological basis for potential convergence in the colorectal microenvironment [15,86,162].

Genotoxic bacteria induce double-strand breaks, activate ATM/ATR-mediated damage response pathways, and stimulate NF-κB-driven inflammation [163]. These same pathways regulate viral episome maintenance, chromatin accessibility, and the transcriptional activity of stress-responsive viral promoters. For example, ATM/ATR activation has been shown to enhance viral gene expression in other epithelial contexts, while NF-κB promotes transcription of lytic or early viral genes in several oncogenic viruses [164,165]. Thus, genotoxin-induced replication stress or inflammatory signaling may provide a permissive environment for viral transcriptional activation in latently infected colorectal epithelial cells. The biological plausibility of cooperative interactions between these microorganisms is supported by their shared capacity to disrupt genomic integrity [166]. Genomic instability and the accumulation of mutations (particularly driver mutations) are central events in the initiation and progression of CRC [167]. It is conceivable that the combined action of bacterial genotoxins and viral factors could synergistically enhance DNA damage, impair repair mechanisms, and accelerate malignant transformation.

Moreover, interaction models described in gastric and hepatic contexts (such as the synergy between *H. pylori* and EBV in GC, or between *Helicobacter hepaticus* and hepatitis B virus in liver carcinogenesis) may offer conceptual frameworks that are, to some extent, adaptable to the colorectal environment [108,168,169,170]. These precedents underscore the need for further research into microbial cooperation in colorectal carcinogenesis, a promising yet underexplored area. Notwithstanding these analogies, the mechanisms proposed for the colon remain hypothetical at present. To rigorously test their relevance in the colorectal microenvironment, future studies should employ (i) human colon organoids and patient-derived tumoroids, (ii) colon organotypic cultures (preserving epithelial polarity, crypt architecture, and stromal–immune context), and (iii) co-culture systems with defined bacterial consortia and relevant viruses, enabling controlled interrogation of epithelial responses, replication stress, and DNA damage signaling [171,172].

**Figure 1:**
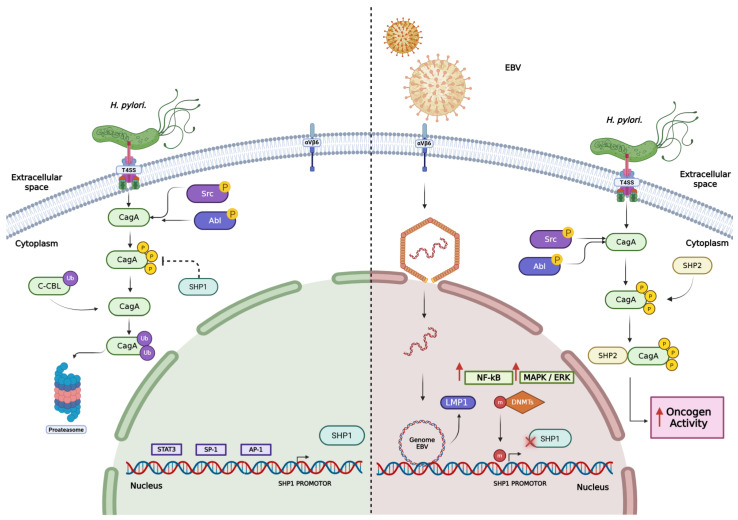
EBV induces hypermethylation of the SHP1 promoter. The *H. pylori* oncoprotein CagA requires phosphorylation to exert its biological function. This phosphorylation is usually removed by the phosphatase SHP1; however, in the presence of EBV, the virus induces hypermethylation of the SHP1 promoter, leading to its epigenetic silencing and allowing CagA to maintain its functional activity, thereby enhancing oncogenic signaling in the host cell. Based on the available literature [156]. Integrin Alpha v Beta 6 (αvβ6). Created in BioRender. Darling Haro. (2025). https://BioRender.com/ (15 December 2025).

Studies have shown that the concomitant presence of EBV and *H. pylori* in the gastric epithelium is associated with a significant increase in the relative risk of developing GC [173]. This phenomenon has been linked, in part, to the ability to induce hypermethylation of the SHP1 phosphatase promoter. Since SHP1 plays a regulatory role by dephosphorylating and inactivating the *H. pylori* oncoprotein CagA, its epigenetic silencing favors the persistence of phosphorylated CagA and, consequently, amplifies the oncogenic signaling driven by this bacterial protein (Figure 1) [156].

As observed, viral and bacterial pathogens can induce diverse mechanisms that damage the host or modify key cellular processes. Therefore, they constitute important risk factors for carcinogenesis by increasing genomic instability and disrupting the host cell cycle. Bacterial pathogens promote genomic damage through various genotoxins, which, over time, are projected to intensify genomic instability [106].

**Figure 2:**
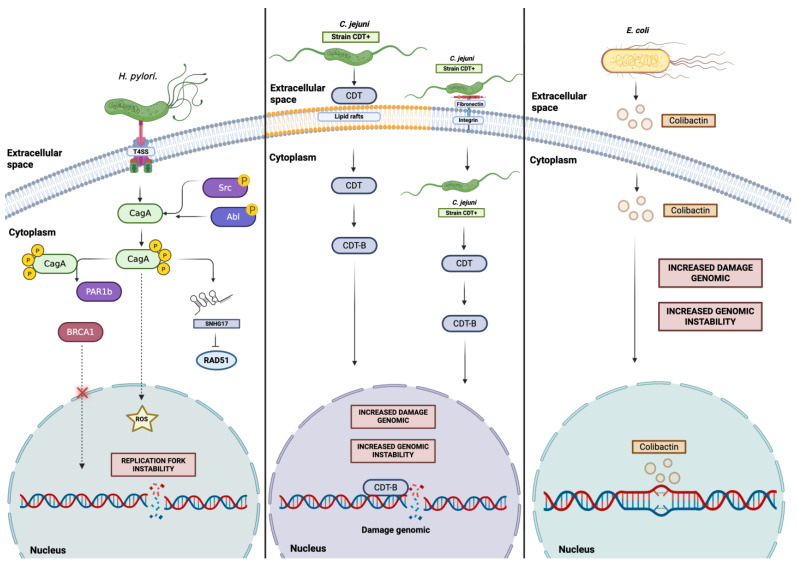
Various mechanisms underlying bacterial-induced cellular stress. The CagA protein induces cellular stress when its concentration increases, thereby activating mechanisms that promote ROS production or the expression of proteins that inhibit DNA repair. By affecting these processes, CagA can alter key effectors of multiple DNA repair pathways. In this context, CagA has been shown to indirectly inhibit BRCA1 and, through downregulation of RAD51 messenger RNA, RAD51 levels in a PAR1b- and SNHG17-dependent manner. In contrast, the catalytic subunit CdtB and the secondary metabolite colibactin induce cellular stress by generating genomic damage in the host cell, thereby contributing to genomic instability. Based on the available literature, we propose the following [106,125,152,153,174,175]. Created in BioRender. Darling Haro. (2025). https://BioRender.com/ (15 December 2025).

To provide a visual synthesis of these processes, Figure 2 summarizes the principal mechanisms through which genotoxic bacteria induce DNA damage, promote replication stress, and disrupt key components of the DNA damage response, ultimately contributing to genomic instability in colorectal epithelial cells (Figure 2).

**Figure 3:**
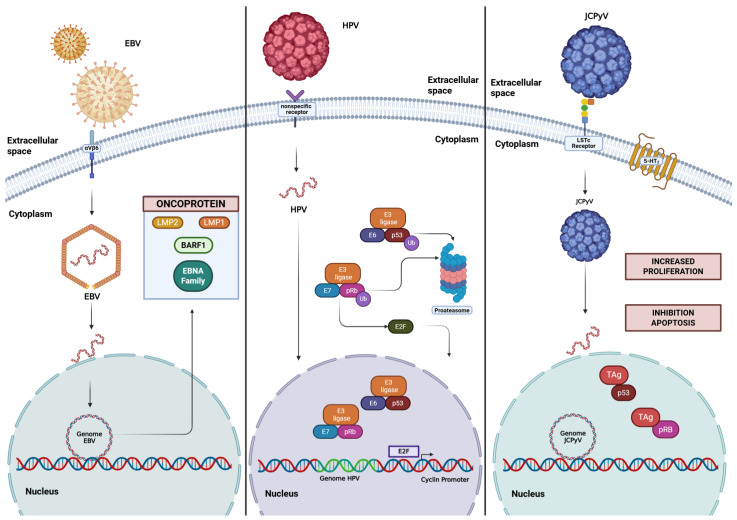
Distinct mechanisms by which viral agents deregulate cellular signaling pathways. EBV, by synthesizing various oncoproteins, can interfere with multiple cellular mechanisms. Similarly, HPV can induce the ubiquitination of p53 and pRb via its E6 and E7 proteins, respectively, thereby inhibiting apoptosis and increasing cellular proliferation. Likewise, JCPyV can inhibit apoptosis and promote cellular proliferation by sequestering p53 and pRb, respectively, through its TAg protein. Based on the available literature, we hypothetically propose the following [9,33,89]. Created in BioRender. Darling Haro. (2025). https://BioRender.com/ (15 December 2025).

On the other hand, viral agents can modulate cellular processes. Figure 3 offers an integrated overview of the major molecular pathways activated by oncogenic viruses in colorectal epithelial cells, highlighting how viral oncoproteins interfere with p53, pRb, cell cycle control, apoptosis, and genome maintenance to promote a pro-transforming cellular environment (Figure 3).

## 5. Conclusions and Future Research

Understanding the potential interactions between viruses and bacteria within the colonic environment is highly relevant to elucidating the role of the colonic microbiota in oncogenesis, as these interactions may help explain why risk factors for CRC remain incompletely defined. Clarifying the significance of these functional interactions among pathogens in the initiation and development of neoplasia, as well as how they may affect cellular processes, could enable the development of novel therapeutic strategies targeting both pathogen types. Such approaches may help prevent molecular and cellular events that promote oncogenesis.

Despite growing interest in microbial contributions to colorectal cancer, many potential interactions between viruses and genotoxic bacteria remain poorly investigated, including several mechanisms discussed in this review. Consequently, our understanding of how these pathogens may induce specific molecular markers associated with colorectal carcinogenesis remains limited.

Nevertheless, the clinical utility of these microbial or viral biomarkers remains uncertain, largely due to the strong dependence of detection outcomes on the analytical methods employed. This variability raises concerns regarding reproducibility and comparability across studies. Prior to proposing therapeutic or diagnostic targets, it is imperative to establish standardized protocols for sample processing, detection thresholds, and validation in multicenter cohorts. Methodological harmonization will be essential to ensure that these markers can transition from experimental observations to clinically actionable tools.

Research on bacterial–viral genotoxic interactions remains largely unexplored, underscoring the need for future studies to validate the proposed mechanisms and assess their biological relevance. More broadly, it is critical to investigate how these interactions may influence key cellular pathways, such as DNA damage responses and cell cycle regulation, as these processes are central to the development of genomic instability and the progression of oncogenic transformation.

This review highlights the main conceptual advances regarding how these microorganisms may contribute to colorectal oncogenesis, with increased genetic damage and dysregulation of cellular signaling pathways emerging as the principal mechanisms. However, these pathogens alone do not appear capable of inducing carcinogenesis. Therefore, in combination, they may constitute a significant risk factor. A substantial proportion of the pathogens discussed in this review are supported by epidemiological data and rigorous statistical analyses. Accordingly, an important association between these pathogens and CRC cannot be excluded. Nevertheless, further epidemiological evidence and well-designed studies providing clear links between these microbial agents and CRC oncogenesis in humans are still required.

Elucidating these unresolved questions will enhance our understanding of how these microbial entities influence the initiation of diseases such as CRC, thereby facilitating insight into the molecular mechanisms that drive oncogenesis. This knowledge may contribute to the prevention of neoplastic development or even lead to tumor regression. Thus, the development of molecular and epidemiological studies to elucidate the contribution of functional interactions mediated by the co-presence of microbial entities in the human colon is imperative.

## Figures and Tables

**Table 1:** Detection of JCPyV in normal tissue and CRC.

Normal Tissue (+JCPyV)	CRC (+JCPyV)	Normal Tissue (+BKPyV)	CRC (+BKPyV)	Method	Reference
N/A	N/A	6%	30%	Nested PCR	[30]
0%	4.3%	4.1%	21.7%	Optimized multiplex PCR	[31]
60%	58.3%	N/A	N/A	Nested PCR	[27]
38%	60%	N/A	N/A	IHC/qPCR	[28]
15.5%	18.6%	N/A	N/A	qPCR	[29]
89%	93%	N/A	N/A	qPCR	[15]

Not available (N/A).

**Table 2:** Detection of HPV in normal tissue, adenoma, and CRC.

Normal Tissue (+HPV)	Adenoma (+HPV)	CRC (+HPV)	Method	Reference
N/A	52%	N/A	PCR	[67]
N/A	N/A	69%	PCR	[68]
16%	37%	N/A	PCR	[69]
14.4%	N/A	N/A	PCR	[70]
35%	36.8%	1.67%	Nested PCR	[71]

Not available (N/A).

**Table 3:** Detection of EBV in normal tissue and CRC.

Normal Tissue (+EBV)	Adenoma (+EBV)	CRC (+EBV)	Method	Reference
16.4%	N/A	51.4%	PCR	[82]
16.4%	N/A	49.1%	IHQ	[83]
N/A	N/A	21%	PCR	[67]
N/A	N/A	28.7%	PCR	[84]
4.1%	N/A	30.5%	ISH/IHC	[85]
N/A	21.1%	18.6%	PCR	[86]

Not available (N/A).

**Table 4:** Main functions of the viral proteins.

Virus	Viral Protein	Interaction	Hallmarks
EBV	EBNA1	Increased mRNA levels of *MDM2* and *MDM4*	Inhibition p53 and apoptosis [12]
EBV	LMP2A	Increased expression of survivin	Inhibition apoptosis [90]
EBV	LMP2A	Increased resistance to apoptosis mediate Bcl-2	Inhibition apoptosis [90]
EBV	LMP2A	Increased stability and translocation of β-catenin	Increase proliferation and metastasis [93]
EBV	LMP1	Increased activation of NF-κB	Increase proliferation/inhibition apoptosis [91]
JCPyV	Antigen T	pRB is sequestered by the T Antigen	Inhibition of apoptosis [16]
JCPyV	Antigen T	p53 is sequestered by the T Antigen	Cell cycle deregulation [16]
JCPyV	Antigen T	β-catenin is translocated to the nucleus by the T Antigen	Increase proliferation and activating metastasis [43,44]
JCPyV	Antigen T	IRS-1 is translocated to the nucleus by the T Antigen	Inhibition of homologous repair [49]
HPV	E6	Induce degradation of p53	Inhibition of apoptosis [9]
HPV	E7	Induce degradation of pRb	Cell cycle deregulation [9]

**Table 5:** Detection of *pks+ E. coli* in normal tissue and CRC.

Normal Tissue (+*E. coli* PKS+)	CRC (+*E. coli* PKS+)	Method	Reference
0%	44%	PCR	[102]
0%	9.2%	PCR	[100]
4.4%	16.7%	PCR	[101]
13%	26%	PCR	[99]
19.3%	55.3%	PCR	[104]

**Table 6:** Detection of *H. pylori* in normal tissue, adenoma, and CRC.

Normal Tissue (+*H pylori*)	Adenoma (+*H pylori*)	CRC (+*H pylori*)	Method	Reference
N/A	N/A	32.9%	Giemsa	[143]
45.6%	30.6%	N/A	immunochromatography	[144]
37.7%	48.6%	N/A	immunohistochemistry	[145]
23.1%	31.9%	N/A	Urea Breath Test	[146]
42.9%	N/A	55%	immunochromatography	[147]

Not available (N/A).

**Table 7:** Main functions of the bacterial proteins.

Bacteria	Toxin	Interaction	Mechanism
*E. coli*	Colibactin	Genetic damage	Alkylations, Crosslinks and replicative stress [105,106]
*E. coli* and *C. jejuni*	CDT	DNA damage	Single and double strand breaks [126]
*C. jejuni*	CDT	Promotes tumorigénesis and metastasis	CDT activate JAK/STAT3/MMP9 pathway [95,139]
*H. pylori*	CagA	Increase proliferation and metastasis	Deregulation of β-catenin [151]
*H. pylori*	VacA	Increase in ROS	Induces greater mitochondrial membrane permeability [157]

## Data Availability

(PDF) Oncolytic Viruses as a Novel Therapeutic Approach for Colorectal Cancer: Mechanisms, Current Advances, and Future Directions. Available from: https://www.researchgate.net/publication/392347328_Oncolytic_Viruses_as_a_Novel_Therapeutic_Approach_for_Colorectal_Cancer_Mechanisms_Current_Advances_and_Future_Directions [accessed on 22 December 2025].

## Abbreviations

The following abbreviations are used in this manuscript:

CRC	Colorectal cancer
JCPyV	Polyomavirus JC
EBV	Epstein–Barr Virus
HPV	Human papillomavirus
HR-HPV	High-risk human papillomavirus
h-TERT	Catalytic subunit of telomerase
TAg	Large T Antigen
NCCR	Non-coding control region
Ori	Origin of replication
PML	Progressive multifocal leukoencephalopathy
NHEJ	Non-homologous end joining
BKPyV	BK virus
pRb	Retinoblastoma protein
NPC	Nasopharyngeal cancer
GC	Gastric cancer
HL	Hodgkin lymphoma
EBNA1	Epstein–Barr nuclear antigen 1
LMP1	Latent membrane protein 1
NF-κB	Nuclear Factor kappa-light-chain-enhancer of activated B cells
CDT	Cytolethal distending toxin
*C. jejuni*	*Campylobacter jejuni*
*E. coli*	*Escherichia coli*
*H. pylori*	*Helicobacter pylori*
IARC	International Agency for Research on Cancer
ICTV	International Committee on Taxonomy of Viruses
ROS	Reactive oxygen species
αvβ6	Integrin Alpha v Beta 6
